# How Creative Self-Regulation Is Associated with Adolescent Creativity: The Sequential Mediating Roles of Cognitive Flexibility and Creative Self-Efficacy

**DOI:** 10.3390/bs16091617

**Published:** 2026-09-10

**Authors:** Xiaofang Xu, Shuo Tang, Tao Chen, Hua Jin

**Affiliations:** 1 Faculty of Psychology, Tianjin Normal University, No. 393, Binshui West Road, Xiqing District, Tianjin 300387, China; xuxiaofang@stu.tjnu.edu.cn (X.X.); 2200340001@stu.tjnu.edu.cn (S.T.); chentao@henau.edu.cn (T.C.); 2 School of Education, Hohhot Minzu College, Hohhot 010051, China; 3 Disciplinary Inspection Commission Agency, Henan Agricultural University, Zhengzhou 450046, China; 4 Key Research Base of Humanities and Social Sciences of the Ministry of Education, Academy of Psychology and Behavior, Tianjin Normal University, Tianjin 300387, China; 5 Tianjin Key Laboratory of Student Mental Health and Intelligence Assessment, Tianjin 300387, China

**Keywords:** creative self-regulation, adolescent creativity, cognitive flexibility, creative self-efficacy, sequential mediation

## Abstract

Understanding the relationship between creative self-regulation and adolescent creativity is important for advancing research on creativity development. However, the psychological processes that may account for this association remain insufficiently explored. The present study aimed to examine the relationship between creative self-regulation and self-reported domain-specific creativity among adolescents, with particular attention to the sequential mediating roles of cognitive flexibility and creative self-efficacy. A total of 1204 Chinese adolescents completed the Creative Self-Regulation Questionnaire (CSRQ), the 20-item Kaufman Domains of Creativity Scale (20-K-DOCS), the Cognitive Flexibility Inventory (CFI), and the Creative Self-Efficacy Scale (CSES). The results showed that creative self-regulation was positively associated with self-reported domain-specific creativity, with significant indirect associations through cognitive flexibility and creative self-efficacy. In addition, a significant sequential indirect association was observed through cognitive flexibility and creative self-efficacy. Specifically, higher levels of creative self-regulation were associated with greater cognitive flexibility, which was in turn associated with higher creative self-efficacy and higher self-reported creativity scores. These findings support an integrated cognitive–motivational framework for understanding the interrelationships among creative self-regulation, cognitive flexibility, creative self-efficacy, and self-reported domain-specific creativity among adolescents, while highlighting these constructs as potentially relevant psychological resources in educational contexts. Given the cross-sectional design, these findings should not be interpreted as establishing causal or temporal relationships among the constructs.

## 1. Introduction

Creativity is increasingly recognized as a core competence for individual development, educational success, and societal innovation in the 21st century. Its importance has become particularly salient as young people are increasingly required to respond to rapid technological change, complex social challenges, and open-ended problems for which established solutions may be insufficient. Creative thinking enables students not only to generate novel and useful ideas, but also to approach problems from multiple perspectives, adapt existing knowledge, and evaluate and refine possible solutions. Accordingly, fostering creativity during adolescence is increasingly viewed as an important educational goal for preparing young people for future learning, work, and participation in a rapidly changing society. Recent scholarship has likewise emphasized that schools can play an important role in shaping children’s and adolescents’ creative development, while also highlighting that the relationship between formal education and creativity is complex and depends on the opportunities, practices, and learning environments provided to students (Rawlings & Cutting, 2024).

This growing emphasis on creativity is also reflected in contemporary international educational policy and assessment. In PISA 2022, the Organisation for Economic Co-operation and Development (OECD) assessed creative thinking among 15-year-old students in 64 countries and economies for the first time, conceptualizing creative thinking as a competence involving the generation, evaluation, and improvement of ideas (OECD, 2024). The inclusion of creative thinking as a distinct assessment domain alongside established academic competencies reflects the growing international recognition of creativity as a developable educational competence rather than a capacity limited to exceptionally creative individuals. More recent analyses of the PISA creative-thinking data have further emphasized the importance of understanding the conditions under which students generate and develop creative ideas across different tasks and domains (OECD, 2025). Together, these developments underscore the growing need to identify psychological and educational resources that may support creativity during adolescence.

Adolescence represents an important period for creativity development, during which creative capacities and the psychological resources relevant to their expression continue to develop. Earlier studies suggested that creative development during adolescence may follow nonlinear patterns, with periods of growth, decline, or stabilization occurring at different ages (Hu et al., 2004; Wo et al., 2009). More recent evidence, however, indicates that adolescent creativity does not necessarily follow a single uniform developmental trajectory. For example, Lin and Shih (2022) found different developmental patterns for open- and closed-ended creative potential among students in Grades 7–11, with open-ended creativity remaining relatively stable across grades while closed-ended creativity showed a more pronounced increase. Using a longitudinal person-centered approach, Anderson (2024) identified five distinct trajectories of divergent-thinking originality across Grades 6–8, with most students showing gradual declines but a smaller proportion maintaining high levels or exhibiting growth. Similarly, Sawada et al. (2024) identified low-, moderate-, and high-creativity trajectories among Japanese junior high school students across three measurement occasions. Together, these findings suggest that creativity development during adolescence is heterogeneous and may vary across individuals, developmental periods, and forms of creativity. Importantly, recent longitudinal evidence also indicates that these developmental differences are associated with potentially malleable individual and contextual factors, including growth-oriented beliefs, engagement, environmental support, and perceived parental autonomy support (Anderson, 2024; Sawada et al., 2024).

Research on adolescent creativity has identified a broad range of individual and contextual correlates, including personality, cognitive and motivational characteristics, and environmental influences. More recently, growing attention has been directed toward the self-regulatory processes involved in creative activity. From this perspective, creativity involves not only generating ideas but also managing uncertainty, sustaining engagement, adjusting strategies, overcoming obstacles, and refining ideas throughout the creative process (Botella et al., 2019; Zielińska et al., 2022). Empirical research has further demonstrated that self-regulatory strategies are associated with the creativity of adolescents’ longer-term projects (Zielińska et al., 2022), while experimental evidence indicates that prompting self-regulatory engagement during creative tasks can enhance creative performance (Zielińska et al., 2025). Nevertheless, comparatively less attention has been paid to the psychological processes that may accompany the association between creative self-regulation and creativity, particularly to how cognitive and motivational resources may operate jointly or sequentially during adolescence.

Against this background, the present study draws primarily on Social Cognitive Theory to examine a theoretically specified sequential mediation framework involving creative self-regulation, cognitive flexibility, creative self-efficacy, and self-reported domain-specific creativity among adolescents. Specifically, we examine whether the observed associations among these constructs are consistent with the proposed sequence from creative self-regulation through cognitive flexibility and creative self-efficacy to creativity. The study contributes to the literature in three respects. First, it extends the emerging literature on creative self-regulation by examining its association with creativity in a large adolescent sample. Second, it integrates cognitive flexibility and creative self-efficacy within a single cognitive–motivational framework to examine how these theoretically relevant resources may jointly account for this association. Third, it provides evidence relevant to understanding how self-regulatory, cognitive, and motivational characteristics are interconnected with adolescents’ self-reported creative functioning in an educational context. Given the cross-sectional design, however, the proposed sequence is treated as a theoretically specified pattern of associations rather than as evidence of temporal or causal ordering.

### 1.1. The Relationship Between Creative Self-Regulation and Creativity

Creative self-regulation refers to the processes through which individuals plan, monitor, adjust, and reflect on their cognition, motivation, emotions, and behavior during creative activity. Unlike conventional tasks with clearly specified goals and solution paths, creative tasks are often ill-defined and characterized by uncertainty, ambiguity, and repeated evaluation and revision. Successfully transforming an initial idea into a creative outcome therefore requires more than idea generation; individuals must also sustain engagement, tolerate uncertainty, monitor progress, modify ineffective strategies, and decide when and how to refine or implement their ideas (Ivcevic & Nusbaum, 2017; Zielińska et al., 2022).

From a social-cognitive perspective, self-regulation provides a mechanism through which individuals actively organize and adapt their behavior in pursuit of personally meaningful goals. Applied to creative activity, this perspective suggests that creative potential is more likely to be translated into creative outcomes when individuals can strategically regulate the different demands that arise across the creative process. Recent research provides empirical support for this view. In a large adolescent sample, Zielińska et al. (2022) identified distinct self-regulatory strategies operating before, during, and after longer-term creative projects and found that these strategies were associated with differences in project creativity. Subsequent studies have further demonstrated the relevance of creative self-regulation across creative activities and contexts. Most notably, experimental evidence showed that prompting individuals to engage in self-regulatory strategies during creative tasks resulted in more creative products than a no-prompt condition (Zielińska et al., 2025).

These findings suggest that creative self-regulation is relevant not only to maintaining engagement in creative activity but also to how ideas are developed and translated into creative outcomes. This relationship may be particularly important during adolescence, when students increasingly encounter open-ended academic and extracurricular tasks that require them to manage goals, strategies, uncertainty, and feedback with greater autonomy. Nevertheless, evidence specifically examining creative self-regulation in adolescent populations remains comparatively limited. Accordingly, the present study examines whether adolescents reporting higher levels of creative self-regulation also report higher levels of domain-specific creativity. We therefore propose the following:

**H1.**  *Creative self-regulation is positively associated with adolescents’ self-reported domain-specific creativity.*

### 1.2. The Mediating Role of Cognitive Flexibility

Cognitive flexibility refers to the capacity to adaptively shift among perspectives, ideas, or strategies in response to changing situational demands. It is particularly relevant to creativity because creative activity often requires individuals to move beyond familiar approaches, reconsider problems from alternative perspectives, and modify strategies when initial attempts prove ineffective (Dennis & Vander Wal, 2010; Diamond, 2013). Accordingly, cognitive flexibility may provide an important cognitive resource that enables individuals to explore alternative possibilities and respond adaptively to uncertainty during creative activity.

Recent research provides increasingly direct evidence for the association between cognitive flexibility and creativity during childhood and adolescence. For example, R.-N. Wang and Chang (2022), in a study of 765 Chinese junior high school students, found that cognitive flexibility was positively associated with creativity and mediated the association between intrinsic motivation and creativity. More recently, Kalaivanan et al. (2026) conducted a scoping review of empirical studies examining cognitive flexibility and creativity among individuals aged 0–18 years. Across the 14 studies included in the review, nine reported significant positive associations between cognitive flexibility and creativity, although substantial heterogeneity in conceptualization and measurement was also identified. The review therefore provides contemporary evidence supporting the relevance of cognitive flexibility to creativity during childhood and adolescence while also indicating that the strength and consistency of this association may depend on how the two constructs are conceptualized and assessed.

Cognitive flexibility may also represent a theoretically relevant cognitive correlate of creative self-regulation. Creative self-regulation requires individuals to monitor ongoing progress, reconsider goals and strategies, and adjust their approaches when obstacles or unexpected information arise. These regulatory demands are conceptually consistent with the adaptive shifting and strategy modification characteristic of cognitive flexibility. Supporting this broader link between regulatory functioning and cognitive flexibility, Nakhostin-Khayyat et al. (2024) found a significant positive association between self-regulation and cognitive flexibility among university students. Although this evidence concerns general rather than creative self-regulation and was obtained from an adult student sample, it provides preliminary empirical support for an association between regulatory functioning and flexible cognitive adjustment. Accordingly, adolescents who report greater capacity to regulate their creative activities may also be more likely to flexibly adjust their thinking and strategies. In turn, greater cognitive flexibility may be associated with higher creativity by enabling adolescents to consider alternative approaches, overcome fixation, and adapt their strategies during open-ended tasks.

Taken together, these theoretical arguments and empirical findings suggest that cognitive flexibility may help account for part of the association between creative self-regulation and adolescent creativity. Given the cross-sectional nature of the present study, this proposed mediation is conceptualized as an indirect association consistent with the hypothesized theoretical ordering rather than as evidence that creative self-regulation causally increases cognitive flexibility. We therefore propose the following:

**H2.**  *Cognitive flexibility mediates the positive association between creative self-regulation and adolescents’ self-reported domain-specific creativity.*

### 1.3. The Mediating Role of Creative Self-Efficacy

Creative self-efficacy (CSE) refers to individuals’ beliefs in their capacity to produce creative outcomes (Tierney & Farmer, 2002). Within Social Cognitive Theory, self-efficacy beliefs influence how individuals approach challenging activities, the effort they invest, and their persistence when difficulties arise (Bandura, 1997, 2001). In creative contexts, such beliefs are particularly relevant because creative activities frequently involve uncertainty, unsuccessful attempts, evaluation, and the possibility of failure. Adolescents with stronger CSE may therefore be more willing to engage with creative challenges, persist when initial ideas are unsuccessful, and continue developing and refining their ideas.

A growing body of evidence supports the relevance of CSE to creativity, including recent research with adolescents. Importantly, Q. Wang et al. (2024) provided longitudinal evidence from 1269 Chinese adolescents showing that creative self-efficacy mediated the longitudinal association between executive functions and everyday creativity; emotional resilience and creative self-efficacy also operated sequentially in this association. More recently, Han et al. (2025) found that creative self-efficacy mediated the association between STEM-specific grit and STEM creativity among Chinese adolescents in Grades 7–9. Together, these findings provide contemporary evidence that CSE is closely associated with adolescents’ creative functioning across different contexts and may represent an important motivational correlate of how cognitive and personal resources are related to creative outcomes.

Creative self-efficacy may also be theoretically linked to both self-regulatory functioning and cognitive flexibility. From a social-cognitive perspective, self-efficacy beliefs are shaped in part by individuals’ interpretations of their experiences of competence and successful task engagement (Bandura, 1997). Self-regulation may contribute to such experiences by helping individuals set goals, monitor progress, adjust strategies, and persist when difficulties arise. Consistent with this broader link, Beeftink et al. (2012) found that self-regulation and domain-specific self-efficacy were meaningfully related within creative professional activity. Although this evidence was obtained from adult creative professionals and concerned general work-related self-regulation rather than adolescent creative self-regulation, it provides indirect empirical support for an association between regulatory functioning and efficacy beliefs in creative contexts.

Cognitive flexibility may also be relevant to CSE. Flexible thinking allows individuals to consider alternative perspectives, revise ineffective strategies, and adapt their responses when circumstances change. Successfully navigating such cognitive demands may provide competence-related information relevant to judgments of creative capability. Recent longitudinal research has linked executive functions with creative self-efficacy among adolescents, although executive functions are conceptually broader than cognitive flexibility (Q. Wang et al., 2024). Thus, cognitive flexibility may represent one cognitive resource associated with stronger confidence in managing creative challenges. Taken together, these theoretical arguments and empirical findings suggest that creative self-efficacy may help account for part of the association between creative self-regulation and adolescent creativity. Given the cross-sectional design of the present study, the proposed mediation is interpreted as an indirect association consistent with the hypothesized theoretical ordering rather than as evidence of temporal or causal effects. We therefore propose the following:

**H3.**  *Creative self-efficacy mediates the positive association between creative self-regulation and adolescents’ self-reported domain-specific creativity.*

### 1.4. The Sequential Mediating Roles of Cognitive Flexibility and Creative Self-Efficacy

The preceding arguments suggest that cognitive flexibility and creative self-efficacy may operate not only as separate correlates of creative self-regulation and creativity but also sequentially within an integrated cognitive–motivational framework. Creative self-regulation involves monitoring progress, adjusting strategies, and responding adaptively to difficulties during creative activity. These regulatory processes are conceptually related to cognitive flexibility, which enables individuals to shift perspectives and modify ineffective strategies when task demands change. Such flexible adjustment may, in turn, provide competence-related experiences that are relevant to the development of efficacy beliefs. From a social-cognitive perspective, successful experiences of managing challenging tasks constitute an important source of self-efficacy beliefs (Bandura, 1997).

Empirical evidence provides indirect support for this proposed cognitive-to-motivational ordering. Among adolescents, cognitive flexibility has been positively associated with academic, social, and emotional self-efficacy (Demirtaş, 2020). More recent longitudinal research with Chinese adolescents has also linked executive functions with creative self-efficacy and everyday creativity (Q. Wang et al., 2024), providing broader evidence that cognitive resources may be related to efficacy beliefs in adolescent creative functioning. Because executive functions are conceptually broader than cognitive flexibility, these findings should be viewed as indirect rather than direct support for the proposed CF-to-CSE pathway. Nevertheless, taken together, the available evidence is consistent with the theoretical proposition that adaptive cognitive resources may be associated with stronger efficacy beliefs.

Integrating these arguments, adolescents who report greater creative self-regulation may also report greater cognitive flexibility in managing creative challenges; greater cognitive flexibility may, in turn, be associated with stronger confidence in their creative capabilities, and stronger creative self-efficacy may be associated with higher self-reported creativity. Accordingly, cognitive flexibility and creative self-efficacy are proposed to constitute a sequential cognitive–motivational pathway linking creative self-regulation with creativity. Given the cross-sectional design, this sequence represents a theoretically specified indirect association and should not be interpreted as evidence of temporal or causal ordering. Thus, we propose the following:

**H4.**  *Cognitive flexibility and creative self-efficacy sequentially mediate the positive association between creative self-regulation and adolescents’ self-reported domain-specific creativity.*

## 2. Method

### 2.1. Participants

Participants were recruited from four junior and senior high schools located in Hohhot, Chifeng, and Tongliao, Inner Mongolia, China. The participating schools were selected through convenience sampling based on accessibility and willingness to participate, including two schools in Hohhot, one school in Tongliao, and one school in Chifeng. Within each participating school, students were recruited from intact classes, and eligible students in the selected classes were invited to participate. Thus, school selection was based on convenience sampling rather than probability-based cluster sampling, whereas student recruitment was organized at the intact-class level.

A total of 1400 questionnaires were initially distributed. Invalid responses were excluded according to the following prespecified criteria: (a) failure on attention-check items (*n* = 95), (b) highly patterned responses or multiple responses to single-choice items (*n* = 55), and (c) more than 10% missing responses (*n* = 46). For cases with no more than 10% missing responses, the corresponding original questionnaires were checked where possible and missing entries were verified and corrected against the original response records. The final analytic dataset contained no missing values on the variables included in the primary analyses. After data screening, 1204 valid questionnaires were retained, yielding an effective response rate of 86.0%.

The final sample comprised 544 male (45.18%) and 660 female (54.82%) adolescents. Of these, 513 (42.61%) were junior high school students and 691 (57.39%) were senior high school students. Gender was coded as 1 = male and 2 = female. Grade level was coded from 1 to 6, corresponding sequentially to the three junior high school grades and the three senior high school grades. For the measurement-invariance analysis, grades 1–3 were grouped as junior high school and grades 4–6 as senior high school. Participants ranged in age from 12 to 18 years (*M* = 15.70, *SD* = 1.80). Participants were recruited from four schools, with school-level sample sizes of 510, 423, 141, and 130, respectively. School identifiers were retained in the analytic dataset, whereas class-level identifiers were not retained. Accordingly, school-level clustering could be descriptively evaluated, but within-class dependence could not be directly estimated.

### 2.2. Measures

#### 2.2.1. Creative Self-Regulation Questionnaire (CSRQ)

Creative self-regulation was assessed using the Chinese adaptation of the Creative Self-Regulation Questionnaire (CSRQ), originally developed by Zielińska et al. (2022). The original CSRQ contains 26 items representing seven regulatory strategies involved in creative activity. Prior to the present study, the questionnaire was translated, culturally adapted, and psychometrically evaluated for use with Chinese adolescents in an independent sample of 1037 senior high school students. Importantly, this adaptation sample was entirely independent of the 1204 adolescents included in the present study.

The Chinese adaptation followed a systematic multistep cross-cultural adaptation procedure. After permission for translation and adaptation was obtained from the original scale developer, two psychology doctoral students independently translated the original items into Chinese. Two graduate students with an English-language background who were unfamiliar with the original questionnaire then independently back-translated the Chinese version into English. An expert panel comprising one professor of psychology, one professor of education, and three psychology doctoral students reviewed the original, translated, and back-translated versions with particular attention to conceptual equivalence, linguistic accuracy, cultural appropriateness, and comprehensibility for Chinese adolescents. Minor wording modifications were made where necessary to improve cultural and developmental appropriateness while preserving the conceptual meaning of the original items. The preliminary Chinese version was subsequently pilot-tested with 60 senior high school students, and their feedback regarding item clarity, comprehensibility, and potential ambiguity was used to finalize the instrument.

The psychometric properties of the adapted questionnaire were subsequently evaluated in the independent adaptation sample (*N* = 1037), which was randomly divided into two subsamples for exploratory factor analysis (*n* = 519) and confirmatory factor analysis (*n* = 518). Item analysis and exploratory factor analysis resulted in a 20-item, five-factor structure comprising Expectation of Uncertainty, Strategy Adjustment, Psychological Self-Adjustment, Willingness to Share and Present Creative Outcomes, and Optimization and Refinement of Creative Solutions. Although the Chinese adaptation yielded five rather than the seven factors identified in the original instrument, the revised structure retained the overarching theoretical organization of creative self-regulation across the preparatory, performance, and reflective phases of creative activity. Thus, the five-factor solution should be understood as an empirically supported integration of closely related regulatory strategies in the Chinese adolescent sample rather than as a redefinition of the overarching construct.

The final confirmatory factor analysis in the independent validation subsample supported the five-factor structure, *χ*^2^/*df* = 2.18, CFI = 0.922, TLI = 0.907, RMSEA = 0.048, 90% CI [0.041, 0.055], and SRMR = 0.053. The total scale demonstrated acceptable internal consistency (Cronbach’s α = 0.798). Cronbach’s α coefficients for the five subscales ranged from 0.621 to 0.766. These findings provided preliminary support for the structural validity and internal consistency of the Chinese CSRQ among senior high school students. In the present study, participants responded to the 20 items on a 7-point Likert scale ranging from 1 (strongly disagree) to 7 (strongly agree), with higher scores indicating higher levels of creative self-regulation. Given that the primary analyses examined creative self-regulation as an overarching construct, the total CSRQ score was used in the subsequent mediation analyses. In the present study, the overall Cronbach’s α coefficient was 0.854.

#### 2.2.2. Kaufman Domains of Creativity Scale (20-K-DOCS)

Self-reported domain-specific creativity was assessed using the 20-item Kaufman Domains of Creativity Scale (20-K-DOCS), developed by Tan et al. (2021). Previous psychometric evaluation of the Chinese 20-K-DOCS was conducted in an adolescent sample aged 15–18 years and supported its five-factor structure and reliability (Cao et al., 2025). The present sample covered a broader adolescent age range (12–18 years); therefore, measurement invariance across junior and senior high school students was additionally evaluated in the present study. The scale consists of 20 items covering five domains of creativity: (a) everyday/interpersonal creativity, (b) scholarly creativity, (c) performance creativity, (d) scientific/mechanical creativity, and (e) artistic creativity. Participants rated each item on a 5-point Likert scale, with higher scores indicating higher levels of self-reported domain-specific creativity. Importantly, the 20-K-DOCS assesses adolescents’ self-reported creative behaviors and self-perceptions across multiple domains rather than objectively assessed or directly observed creative performance. In the present study, the scale demonstrated good internal consistency, with a Cronbach’s α of 0.897.

#### 2.2.3. Cognitive Flexibility Inventory (CFI)

Cognitive flexibility was measured using the Cognitive Flexibility Inventory (CFI) developed by Dennis and Vander Wal (2010). The Chinese version adapted by Y. Wang et al. (2016) was used in this study. The inventory consists of 20 items assessing two dimensions: (a) alternatives and (b) control. The “alternatives” dimension reflects the ability to perceive multiple explanations and solutions for difficult situations, whereas the “control” dimension reflects the tendency to perceive challenging situations as manageable. Items were rated on a 5-point Likert scale, with higher scores indicating greater cognitive flexibility. The Chinese version has demonstrated good reliability and validity in Chinese students (Y. Wang et al., 2016). In the present study, Cronbach’s α for the total scale was 0.874.

#### 2.2.4. Creative Self-Efficacy Scale (CSES)

Creative self-efficacy was measured using the Creative Self-Efficacy subscale from the Short Scale of Creative Self (SSCS) developed by Karwowski et al. (2018). The subscale consists of six items rated on a 5-point Likert scale, with higher scores indicating stronger beliefs in one’s ability to produce creative outcomes. In this study, the scale demonstrated good reliability, with a Cronbach’s α coefficient of 0.853.

### 2.3. Procedure

Data collection was conducted in March 2026. The study was conducted in classroom settings with the assistance of trained teachers and researchers. Before data collection, informed consent was obtained from participants and their guardians. Participants were informed that their responses would remain anonymous and confidential and would be used for research purposes only. The questionnaires were completed voluntarily during school hours and took approximately 20–25 min to finish.

This study was approved by the Ethics Committee of Tianjin Normal University (Approval No. 2025110303). All procedures involving human participants were conducted in accordance with the ethical standards of the institutional research committee and the Declaration of Helsinki. Written informed consent was obtained from all participants and their legal guardians prior to participation.

### 2.4. Data Analysis

Data analyses were conducted using SPSS 27.0, Mplus 8.3, and Hayes’ PROCESS macro for SPSS. Harman’s single-factor test was first used to assess potential common method bias, followed by descriptive statistics and Pearson correlations among the study variables. Measurement-model analyses were conducted in Mplus 8.3 using robust maximum likelihood estimation (MLR), which provides standard errors and a chi-square test statistic that are robust to non-normality. Model evaluation included overall fit, standardized factor loadings, composite reliability (CR), average variance extracted (AVE), discriminant validity, and comparison with a theoretically plausible alternative measurement model.

Because the previous psychometric evaluation of the Chinese 20-K-DOCS was conducted among adolescents aged 15–18 years, whereas the present sample ranged from 12 to 18 years, measurement invariance across junior and senior high school students was additionally examined using multigroup CFA. Configural, metric, and scalar invariance were sequentially tested, with changes in CFI, RMSEA, and SRMR used to evaluate invariance across increasingly constrained models. Given that students were recruited from intact classes within four schools, school-level intraclass correlation coefficients [ICC] were also calculated for the four focal variables to descriptively assess between-school clustering. Class-level ICCs could not be estimated because class identifiers were not retained. Given the small number of schools, the school-level ICCs were interpreted descriptively rather than used as a basis for multilevel or cluster-robust inference.

Finally, Hayes’ PROCESS Model 6 in SPSS 27.0 was used as the primary analysis to test the hypothesized sequential mediation model using observed composite scores. Gender and grade level were included as covariates to account for potential demographic variation associated with adolescent development and creativity-related characteristics. Grade level, rather than chronological age, was used as the developmental covariate because the school-based educational context was organized by grade level, which directly reflects students’ educational stage. Heteroscedasticity-consistent standard errors (HC3) were used for regression coefficients, and the significance of indirect effects was evaluated using percentile bootstrap confidence intervals based on 5000 resamples. An indirect effect was considered statistically significant when its 95% bootstrap confidence interval did not include zero.

## 3. Results

### 3.1. Common Method Bias Test

Because all data were collected using self-report questionnaires, common method bias may have influenced the results. Harman’s single-factor test was conducted to examine this issue. The results showed that 12 factors had eigenvalues greater than one, accounting for 55.76% of the total variance. The first factor explained 23.07% of the variance, which was substantially below the commonly used threshold of 40% (Tang & Wen, 2020). However, Harman’s single-factor test is a limited diagnostic and cannot rule out common method variance (Zhou & Long, 2004). Accordingly, potential common method effects should still be considered when interpreting the observed associations.

### 3.2. Descriptive Statistics and Correlation Analysis

Descriptive statistics and Pearson correlation coefficients for the main variables are presented in Table 1. The results showed that creative self-regulation was significantly and positively correlated with cognitive flexibility (*r* = 0.59, *p* < 0.01), creative self-efficacy (*r* = 0.47, *p* < 0.01), and self-reported domain-specific creativity (hereafter referred to as creativity; *r* = 0.42, *p* < 0.01). Cognitive flexibility was also positively correlated with creative self-efficacy (*r* = 0.60, *p* < 0.01) and creativity (*r* = 0.43, *p* < 0.01). In addition, creative self-efficacy was positively associated with creativity (*r* = 0.53, *p* < 0.01). These findings provided preliminary support for the hypothesized relationships among the variables.

Given the nested sampling structure, school-level ICC values were calculated for the four focal variables. The ICCs were 0.049 for creative self-regulation, 0.073 for cognitive flexibility, 0.032 for creative self-efficacy, and 0.028 for self-reported domain-specific creativity. Thus, between-school variation in the focal variables was relatively limited, with school-level clustering accounting for approximately 2.8% to 7.3% of the total variance. However, these estimates were interpreted descriptively because only four schools participated in the study. Class-level clustering could not be evaluated because class identifiers were not retained in the final dataset.

### 3.3. Measurement Model

A confirmatory factor analysis was first conducted to examine the measurement structure and empirical distinctiveness of the focal constructs. The hypothesized multidimensional measurement model comprised five first-order factors for creative self-regulation, two factors for cognitive flexibility, one factor for creative self-efficacy, and five domain-specific factors for self-reported creativity. The model yielded *χ*^2^ = 4671.769, *df* = 2001, *χ*^2^/*df* = 2.33, CFI = 0.898, TLI = 0.891, RMSEA = 0.033, 90% CI [0.032, 0.035], and SRMR = 0.056. The RMSEA and SRMR indicated good absolute fit, while the CFI and TLI were close to, but slightly below, the conventional 0.90 criterion. Overall, the fit indices suggested an acceptable, although not uniformly strong, model fit.

The measurement properties of the first-order factors were further evaluated using standardized factor loadings, composite reliability (CR), average variance extracted (AVE), and HTMT. For concise reporting, the ranges of standardized factor loadings, CR and AVE estimates, and the maximum HTMT coefficient for each first-order factor are provided in Supplementary Table S1. Measurement quality varied across the first-order factors, with several subdimensions showing comparatively low AVE values. Accordingly, the results should not be interpreted as indicating uniformly strong convergent validity across all first-order factors.

In addition, because the present sample covered a broader adolescent age range than that included in the previous Chinese validation study of the 20-K-DOCS, measurement invariance across junior and senior high school students was examined using multigroup confirmatory factor analysis (see Supplementary Table S2). The configural model showed an acceptable fit, *χ*^2^(320) = 816.185, CFI = 0.937, TLI = 0.926, RMSEA = 0.051, 90% CI [0.046, 0.055], and SRMR = 0.048. Metric invariance was supported after constraining factor loadings to equality across groups (|ΔCFI| = 0.003, |ΔRMSEA| < 0.001, |ΔSRMR| = 0.004), and scalar invariance was further supported after constraining item intercepts to equality (|ΔCFI| = 0.004, |ΔRMSEA| < 0.001, |ΔSRMR| = 0.003). Taken together, these findings support measurement comparability of the five-factor 20-K-DOCS across junior and senior high school students in the present sample.

Of particular relevance to the potential conceptual overlap between creative self-efficacy and self-reported creativity, creative self-efficacy demonstrated satisfactory measurement properties, with standardized factor loadings ranging from 0.703 to 0.791, CR = 0.886, and AVE = 0.564. The HTMT coefficients between creative self-efficacy and interpersonal, academic, performance, scientific, and visual arts creativity were 0.626, 0.560, 0.399, 0.402, and 0.487, respectively. All of these values were below the conventional HTMT thresholds of 0.85 or 0.90 (Henseler et al., 2015), providing evidence that creative self-efficacy was empirically distinguishable from the five domains of self-reported creativity.

To further examine the discriminant validity between creative self-efficacy and self-reported domain-specific creativity, an alternative measurement model was estimated in which the six CSE items and the 20 creativity items were specified to load on a single common factor, while the remaining measurement structure was unchanged. As shown in Table 2, this alternative model showed substantially poorer fit, *χ*^2^(2051) = 9007.469, *χ*^2^/*df* = 4.39, CFI = 0.734, TLI = 0.722, RMSEA = 0.053, 90% CI [0.052, 0.054], and SRMR = 0.076. In addition, its AIC (220,889.187) and BIC (222,040.296) values were considerably higher than those of the hypothesized 13-factor model (AIC = 215,705.880; BIC = 217,111.660). Together with the HTMT results, these findings support the empirical distinctiveness of creative self-efficacy and self-reported domain-specific creativity.

### 3.4. Sequential Mediation Analysis

**Figure 1:**
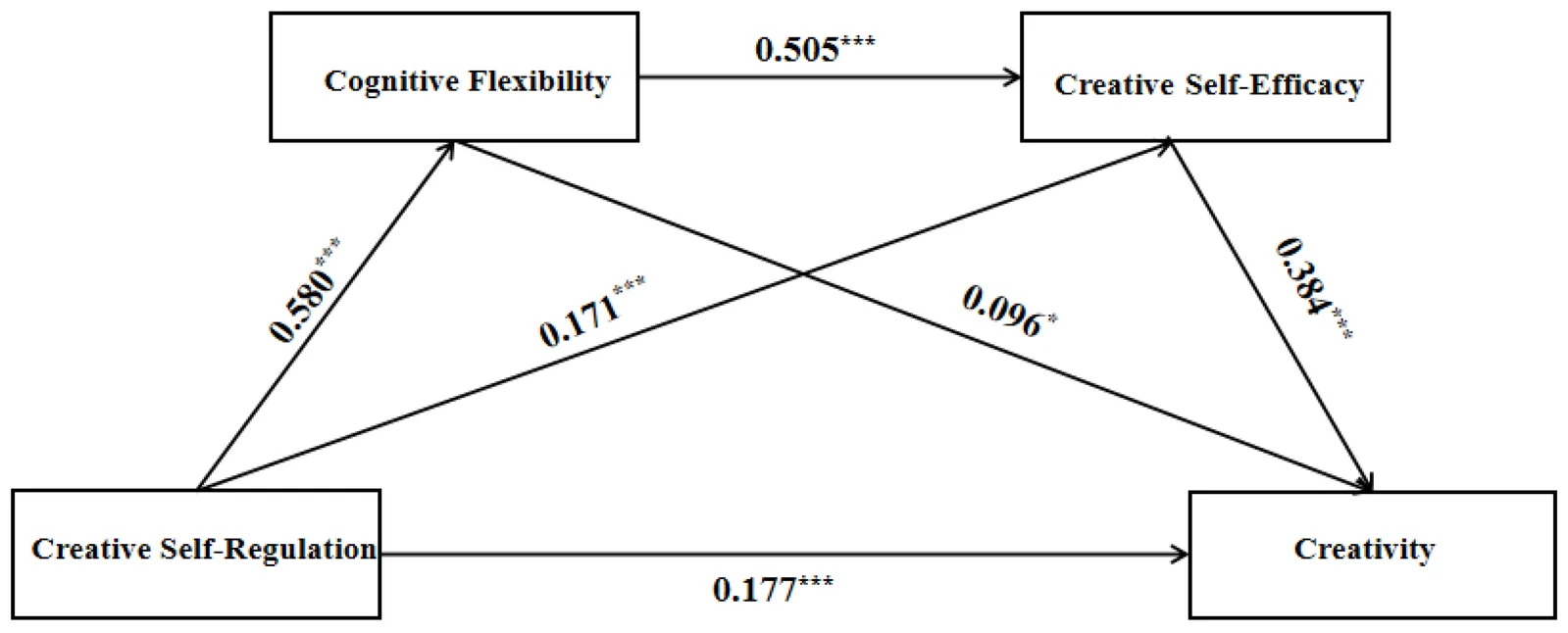
Standardized Path Coefficients for the Hypothesized Sequential Mediation Model. Note. Standardized regression coefficients obtained from PROCESS Model 6 are presented. Gender and grade level were included as covariates but are not shown for clarity. * *p* < 0.05, *** *p* < 0.001.

Following the evaluation of the measurement model, the hypothesized sequential mediation model was tested using Hayes’ PROCESS Model 6, which served as the primary analysis for hypothesis testing. Observed composite scores were used for creative self-regulation, cognitive flexibility, creative self-efficacy, and self-reported domain-specific creativity, with gender and grade level included as covariates. Percentile bootstrap confidence intervals based on 5000 resamples were used to evaluate the statistical significance of the indirect effects. For ease of comparison across paths, standardized regression coefficients are presented in Figure 1, whereas the corresponding inferential statistics are reported using unstandardized coefficients (B).

The total effect of creative self-regulation on creativity was significant (*B* = 0.357, SE = 0.024, *p* < 0.001). After cognitive flexibility and creative self-efficacy were included as mediators, the direct effect of creative self-regulation on creativity remained significant (*B* = 0.154, *SE* = 0.027, *p* < 0.001). Creative self-regulation was positively associated with cognitive flexibility (*B* = 0.423, *SE* = 0.020, *p* < 0.001) and creative self-efficacy (*B* = 0.053, *SE* = 0.010, *p* < 0.001). Cognitive flexibility was positively associated with both creative self-efficacy (*B* = 0.214, *SE* = 0.012, *p* < 0.001) and creativity (*B* = 0.114, *SE* = 0.045, *p* = 0.011), while creative self-efficacy was also positively associated with creativity (*B* = 1.080, *SE* = 0.101, *p* < 0.001). The detailed regression results are presented in Table 3 and the standardized path coefficients are shown in Figure 1.

The statistical significance and magnitude of the specific and total indirect effects were evaluated using percentile bootstrap confidence intervals and are reported in Table 4. The indirect association through cognitive flexibility was significant (*B* = 0.048, Boot*SE* = 0.019, 95% CI [0.011, 0.087]), accounting for 13.45% of the total effect. The indirect association through creative self-efficacy was also significant (*B* = 0.057, Boot*SE* = 0.012, 95% CI [0.034, 0.083]), accounting for 15.97% of the total effect. In addition, the sequential indirect association through cognitive flexibility and creative self-efficacy was significant (*B* = 0.098, Boot*SE* = 0.012, 95% CI [0.075, 0.123]), accounting for 27.45% of the total effect. The total indirect effect was significant (*B* = 0.203, Boot*SE* = 0.021, 95% CI [0.164, 0.245]), accounting for 56.86% of the total effect.

## 4. Discussion

Grounded in Social Cognitive Theory, the present study examined the relationship between creative self-regulation and self-reported domain-specific creativity among adolescents and further tested the mediating roles of cognitive flexibility and creative self-efficacy. The findings showed that creative self-regulation was positively associated with self-reported domain-specific creativity and that significant indirect associations were observed through cognitive flexibility, creative self-efficacy, and their sequential pathway. These findings supported all proposed hypotheses and were consistent with the theoretically specified sequential mediation model. However, given the cross-sectional design, the observed pathways should be interpreted as patterns of association rather than evidence of temporal or causal ordering.

### 4.1. The Relationship Between Creative Self-Regulation and Adolescent Creativity

The results showed that creative self-regulation was significantly and positively associated with adolescents’ self-reported domain-specific creativity, supporting Hypothesis 1. This finding is consistent with earlier work identifying creative self-regulation as an important correlate of creative activity and performance (Zielińska et al., 2022). More recent studies have further strengthened this perspective. For example, Zielińska et al. (2024) showed that self-beliefs and self-regulatory processes were closely related to creative activity among teachers, while Zielińska et al. (2025) provided experimental evidence that self-regulation prompts can improve creative performance. Taken together, these studies highlight the relevance of self-regulatory processes, in addition to idea generation, for understanding how creative activity is sustained and developed over time.

The present findings extend this emerging literature by providing evidence from an adolescent sample. Creative activities are often characterized by openness, uncertainty, and the need for persistence, requiring individuals to regulate goals, adjust strategies, monitor progress, and manage emotional responses throughout the creative process. Adolescents reporting higher levels of creative self-regulation may therefore also be more likely to sustain engagement, adapt strategies, and continue developing ideas when creative tasks become difficult or ambiguous. These characteristics may help explain why creative self-regulation was positively associated with self-reported domain-specific creativity in the present study.

From the perspective of Social Cognitive Theory, self-regulation reflects an agentic process through which individuals organize and adapt their behavior in pursuit of goals. The present results are consistent with this perspective and suggest that creative self-regulation may represent an important self-regulatory resource associated with adolescents’ creative functioning. At the same time, because the present study was cross-sectional, the observed association should not be interpreted as evidence that creative self-regulation causally increases creativity.

### 4.2. The Mediating Role of Cognitive Flexibility

The results showed that cognitive flexibility significantly mediated the positive association between creative self-regulation and self-reported domain-specific creativity, supporting Hypothesis 2. This finding is consistent with earlier research identifying cognitive flexibility as an important correlate of creative thinking and performance (Zabelina & Robinson, 2010; De Dreu et al., 2011). More recent evidence has extended this relationship to school-aged and adolescent populations. R.-N. Wang and Chang (2022), for example, found that cognitive flexibility was positively associated with creativity among Chinese junior high school students. More recently, Kalaivanan et al. (2026), in a scoping review of children and adolescents, reported that most included studies found significant positive associations between cognitive flexibility and creativity, although the strength of the relationship varied depending on how the two constructs were conceptualized and measured.

The present findings further suggest that cognitive flexibility may represent one cognitive correlate through which creative self-regulation is associated with creativity. Creative self-regulation involves monitoring progress, reconsidering strategies, and adapting behavior when difficulties arise, while cognitive flexibility reflects the capacity to shift perspectives and modify ineffective approaches. These processes are conceptually related, and recent research has also reported a positive association between general self-regulation and cognitive flexibility (Nakhostin-Khayyat et al., 2024). Accordingly, adolescents who report stronger creative self-regulation may also report greater flexibility in adapting their thinking and problem-solving strategies.

Greater cognitive flexibility, in turn, may be relevant to creativity because it allows individuals to consider alternative perspectives, overcome fixation, and adjust their approach when initial solutions prove ineffective. Thus, the observed indirect association through cognitive flexibility is consistent with a cognitive pathway linking creative self-regulation and creativity. However, given the cross-sectional design, the present findings do not establish that creative self-regulation precedes or causes changes in cognitive flexibility.

### 4.3. The Mediating Role of Creative Self-Efficacy

The results showed that creative self-efficacy significantly mediated the positive association between creative self-regulation and adolescents’ self-reported domain-specific creativity, supporting Hypothesis 3. This finding is consistent with Social Cognitive Theory, which emphasizes the importance of efficacy beliefs in shaping individuals’ engagement, persistence, and responses to challenging tasks (Bandura, 1997). In creative contexts, confidence in one’s creative capability may be particularly relevant because creative activity frequently involves uncertainty, unsuccessful attempts, evaluation, and repeated revision. Adolescents with stronger creative self-efficacy may therefore be more willing to persist with challenging creative activities and continue developing their ideas when initial attempts are unsuccessful.

The present finding is also consistent with recent research highlighting the role of creative self-efficacy in adolescent creativity. Using a three-wave longitudinal design with 1269 Chinese adolescents, Q. Wang et al. (2024) found that creative self-efficacy mediated the longitudinal association between executive functions and everyday creativity. Similarly, Han et al. (2025) found that STEM-specific creative self-efficacy mediated the association between STEM-specific grit and STEM creativity among Chinese adolescents in Grades 7–9. Although these studies examined different antecedents and forms of creativity, together they provide contemporary evidence that creative self-efficacy is closely associated with adolescent creative functioning across different contexts.

The present study extends this literature by linking creative self-efficacy specifically to creative self-regulation. Adolescents who are better able to set creative goals, monitor their progress, adjust strategies, and persist through difficulties may also develop more positive judgments of their capacity to deal with creative challenges. In turn, stronger creative self-efficacy may be associated with greater willingness to sustain effort and engage with uncertain or demanding creative activities. Thus, the observed indirect association through creative self-efficacy is consistent with a motivational pathway linking creative self-regulation with self-reported creativity. Nevertheless, because the present data are cross-sectional, the findings do not establish that creative self-regulation temporally precedes or causes changes in creative self-efficacy.

### 4.4. The Sequential Mediating Roles of Cognitive Flexibility and Creative Self-Efficacy

The results further showed a significant sequential indirect association through cognitive flexibility and creative self-efficacy, supporting Hypothesis 4. This finding suggests that the association between creative self-regulation and self-reported creativity may involve interconnected cognitive and motivational correlates rather than either cognitive flexibility or creative self-efficacy alone. Specifically, adolescents reporting stronger creative self-regulation also tended to report greater cognitive flexibility; greater cognitive flexibility was associated with stronger creative self-efficacy; and stronger creative self-efficacy was, in turn, associated with higher self-reported domain-specific creativity.

This pattern is consistent with a cognitive–motivational interpretation of adolescent creative functioning. Creative self-regulation involves monitoring ongoing activity and modifying strategies in response to obstacles or changing demands. Cognitive flexibility may facilitate such adaptive adjustment by enabling adolescents to reconsider ineffective approaches and explore alternative perspectives. Successfully navigating these cognitive demands may provide competence-related experiences that are relevant to judgments of creative capability, thereby linking flexible cognition with creative self-efficacy. Stronger creative self-efficacy, in turn, may support continued engagement and persistence when creative activities involve uncertainty or unsuccessful attempts.

Recent evidence provides additional support for the plausibility of this cognitive-to-motivational ordering. Chang et al. (2026), using both survey and experimental approaches, found that cognitive flexibility and creative self-efficacy sequentially mediated the association between awe and creativity. Although their study examined a different antecedent, the ordering of the two mediators is consistent with the CF-to-CSE sequence observed in the present study. Related research among Chinese college students has also reported a sequential indirect pathway involving cognitive flexibility and creative self-efficacy in relation to innovative behavior (Yu et al., 2025). These findings provide converging, although not directly equivalent, evidence that cognitive flexibility and creative efficacy beliefs may be interconnected in creativity-related processes.

The present study extends this emerging evidence to creative self-regulation and adolescent domain-specific creativity. Importantly, however, the significant sequential indirect effect should not be interpreted as demonstrating that cognitive flexibility temporally precedes creative self-efficacy. Because all focal variables were assessed concurrently, alternative directional or reciprocal relationships remain plausible. Longitudinal and experimental studies are therefore needed to test whether changes in creative self-regulation are followed by changes in cognitive flexibility and creative self-efficacy and, ultimately, by changes in creative outcomes.

### 4.5. Implications and Limitations

From a practical perspective, the findings suggest several classroom practices that may be relevant to supporting adolescent creativity. First, teachers may scaffold creative self-regulation by encouraging students to set explicit creative goals before open-ended tasks, monitor their progress during idea development, and reflect on the effectiveness of their strategies after task completion. For example, students could complete brief planning prompts before a project and use reflection sheets to identify which strategies helped or hindered their creative process.

Second, cognitive flexibility may be exercised through classroom activities that require students to generate multiple solutions, reinterpret a problem from different perspectives, compare alternative strategies, or revise an initial solution after receiving new information. Such practices may be incorporated into project-based learning, inquiry tasks, writing activities, scientific problem solving, and interdisciplinary assignments.

Third, creative self-efficacy may be supported by providing students with attainable but challenging creative tasks, opportunities to experience incremental success, process-focused feedback, and exposure to diverse peer models. Rather than emphasizing only the originality of final products, teachers may reinforce students’ willingness to experiment, revise ideas, and persist through unsuccessful attempts. These practices may help students develop greater confidence in their ability to engage successfully with creative challenges.

Importantly, these recommendations should be interpreted as theoretically informed educational implications rather than as evidence of intervention effectiveness. Because the present study was cross-sectional, future experimental and longitudinal research is needed to determine whether classroom practices targeting creative self-regulation, cognitive flexibility, and creative self-efficacy lead to subsequent improvements in creative behavior or objectively assessed creative performance.

Despite these contributions, several limitations should be acknowledged. First, the cross-sectional design precludes conclusions regarding temporal precedence or causality among creative self-regulation, cognitive flexibility, creative self-efficacy, and creativity. Although the observed associations were consistent with the theoretically proposed sequential mediation model, alternative directional, reverse, or reciprocal relationships remain plausible. For example, adolescents who perceive themselves as more creative may also report stronger creative self-efficacy and greater engagement in creative self-regulation. Future research should employ longitudinal cross-lagged designs, intensive longitudinal approaches, or experimental interventions to examine the temporal ordering and potential reciprocal relationships among these constructs.

Second, the sampling and nested data structure should be considered when interpreting the findings. Participating schools were selected through convenience sampling, and students were recruited from intact classes within these schools. School-level ICCs for the four focal variables ranged from 0.028 to 0.073, suggesting relatively limited between-school clustering. However, only four schools participated in the study, precluding reliable school-level multilevel or cluster-robust inference. More importantly, class-level identifiers were not retained in the final dataset; consequently, potential within-class dependence could not be directly quantified or statistically adjusted. Because students within the same class may share teachers, classroom environments, peer contexts, and educational experiences, unmodeled within-class dependence may have affected the estimated standard errors. The statistical significance of the present individual-level associations should therefore be interpreted with this limitation in mind. Future research should recruit participants from a larger number of schools and classes, retain identifiers at each sampling level, and explicitly account for the hierarchical data structure using multilevel modeling or appropriate complex-sampling procedures.

Third, creativity was assessed using the self-report 20-K-DOCS rather than an objective or directly observed measure of creative performance. Accordingly, the outcome in the present study should be interpreted as self-reported domain-specific creativity rather than objective creative performance. This distinction is particularly relevant to the observed association between creative self-efficacy and creativity because both constructs involve individuals’ self-evaluations in the creative domain. Their association may therefore partly reflect conceptual overlap or a broader tendency toward positive self-evaluation. Although creative self-efficacy concerns beliefs about one’s capability to perform creatively, whereas the 20-K-DOCS assesses self-reported creative behaviors and self-perceptions across specific domains, the possibility of shared method and conceptual variance cannot be excluded. Future studies should incorporate multiple methods of creativity assessment, including divergent-thinking tasks, creative product evaluations, behavioral indicators, or ratings by teachers and peers, to examine whether the present pattern of associations generalizes to objectively assessed creative performance.

## 5. Conclusions

In conclusion, the present study provides evidence that creative self-regulation is positively associated with adolescents’ self-reported domain-specific creativity and that cognitive flexibility and creative self-efficacy statistically account for part of this association, both independently and sequentially. These findings highlight the potential relevance of integrating self-regulatory, cognitive, and motivational perspectives when examining adolescent creative functioning. Future research should employ longitudinal and experimental designs to test the temporal ordering and potential causal and reciprocal relationships among these constructs and should incorporate multiple indicators of creativity, including performance-based assessments and reports from teachers or peers. It would also be valuable to examine whether these associations vary across developmental stages, gender, cultural contexts, and educational settings and whether interventions targeting creative self-regulation, cognitive flexibility, or creative self-efficacy can produce measurable improvements in adolescents’ creative functioning.

## Figures and Tables

**Table 1:** Means, standard deviations, and correlations among the study variables (*N* = 1204).

Variable	*M*	*SD*	1	2	3	4
1 Creative self-regulation	100.27	14.86	1			
2 Cognitive flexibility	69.23	10.82	0.59 **	1		
3 Creative self-efficacy	19.86	4.59	0.47 **	0.60 **	1	
4 Creativity	63.86	12.90	0.42 **	0.43 **	0.53 **	1

Note. Creativity refers to self-reported domain-specific creativity assessed using the 20-K-DOCS. ** *p* < 0.01.

**Table 2:** Comparison of the Hypothesized and Alternative Measurement Models.

Model	*χ* ^2^	*df*	CFI	TLI	RMSEA	SRMR	AIC	BIC
Hypothesized multidimensional measurement model	4671.769	2001	0.898	0.891	0.033	0.056	215,705.880	217,111.660
CSE–creativity merged model	9007.469	2051	0.734	0.722	0.053	0.076	220,889.187	222,040.296

Note. CSE = creative self-efficacy; CFI = comparative fit index; TLI = Tucker–Lewis index; RMSEA = root mean square error of approximation; SRMR = standardized root mean square residual; AIC = Akaike information criterion; BIC = Bayesian information criterion. Lower AIC and BIC values indicate better relative model fit.

**Table 3:** Regression Results for the Sequential Mediation Model.

Outcome	Predictor	*B*	*SE* (HC3)	*t*	*p*	95% CI	*β*	*R* ^2^	*F* (HC3)
CF	CSR	0.423	0.020	21.538	<0.001	[0.384, 0.461]	0.580	0.365	166.857 ***
	Gender	−0.794	0.506	−1.569	0.117	[−1.783, 0.199]	−0.037		
	Grade	−0.653	0.142	−4.596	<0.001	[−0.932, −0.374]	−0.108		
CSE	CSR	0.053	0.010	5.121	<0.001	[0.033, 0.073]	0.171	0.383	186.631 ***
	CF	0.214	0.012	17.180	<0.001	[0.190, 0.239]	0.505		
	Gender	0.303	0.212	1.430	0.153	[−0.113, 0.720]	0.033		
	Grade	0.065	0.057	1.131	0.258	[−0.048, 0.177]	0.025		
Creativity	CSR	0.154	0.027	5.615	<0.001	[0.100, 0.208]	0.177	0.320	104.689 ***
	CF	0.114	0.045	2.543	0.011	[0.026, 0.202]	0.096		
	CSE	1.080	0.101	10.738	<0.001	[0.883, 1.277]	0.384		
	Gender	1.281	0.630	2.034	0.042	[0.045, 2.517]	0.049		
	Grade	−0.049	0.173	−0.284	0.776	[−0.390, 0.291]	−0.007		
Creativity (total-effect model)	CSR	0.357	0.024	14.907	<0.001	[0.310, 0.405]	0.412	0.176	78.130 ***
	Gender	1.334	0.688	1.938	0.053	[−0.017, 2.685]	0.051		
	Grade	−0.205	0.187	−1.093	0.274	[−0.573, 0.163]	−0.028		

Note. *** *p* < 0.001; CSR = creative self-regulation; CF = cognitive flexibility; CSE = creative self-efficacy. Creativity refers to self-reported domain-specific creativity assessed using the 20-K-DOCS. *B* = unstandardized regression coefficient; *β* = standardized regression coefficient; *SE*(HC3) = heteroscedasticity-consistent standard error; CI = 95% confidence interval.

**Table 4:** Bootstrap results for indirect effects.

Pathway	Indirect Effect (*B*)	Boot*SE*	95% Bootstrap CI	Effect Ratio (%)
CSR → CF → Creativity	0.048	0.019	[0.011, 0.087]	13.45%
CSR → CSE → Creativity	0.057	0.012	[0.034, 0.083]	15.97%
CSR → CF → CSE → Creativity	0.098	0.012	[0.075, 0.123]	27.45%
Total indirect effect	0.203	0.021	[0.164, 0.245]	56.86%

Note. CSR = creative self-regulation; CF = cognitive flexibility; CSE = creative self-efficacy. Creativity refers to self-reported domain-specific creativity assessed using the 20-K-DOCS. *B* = unstandardized indirect effect; Boot*SE* = bootstrap standard error; CI = percentile bootstrap confidence interval. Effect ratios were calculated by dividing each unstandardized indirect effect by the unstandardized total effect (*B* = 0.357). Minor discrepancies in summed percentages may occur due to rounding.

## Data Availability

Data will be available on request.

## Supplementary Materials

The following supporting information can be downloaded at: https://www.mdpi.com/article/10.3390/bs16091617/s1, Table S1: Measurement Properties of the First-Order Factors; Table S2: Measurement Invariance of the 20-K-DOCS Across Junior and Senior High School Students.
