# Supplementary material for: How Creative Self-Regulation Is Associated with Adolescent Creativity: The Sequential Mediating Roles of Cognitive Flexibility and Creative Self-Efficacy
Source: Behav Sci (Basel). 2026 Sep 10;16(9):1617. doi: 10.3390/bs16091617 (PMC13603239; doi:10.3390/bs16091617)
Supplement: Supplementary file 1 [file behavsci-16-01617-s001.zip › behavsci-4440161-supplementary.pdf]

# Supplementary Materials

## How Creative Self-Regulation Is Associated With Adolescent Creativity: The Sequential Mediating Roles of Cognitive Flexibility and Creative Self-Efficacy

Supplementary Table S1. Measurement Properties of the First-Order Factors

| Factor | Standardized loading range | CR | AVE | Maximum HTMT | Corresponding factor |
|---|---|---|---|---|---|
| CSR-F1 | .463–.714 | .651 | .325 | .352 | CF-Control |
| CSR-F2 | .655–.768 | .839 | .511 | .794 | CSR-F3 |
| CSR-F3 | .484–.663 | .738 | .364 | .794 | CSR-F2 |
| CSR-F4 | .487–.617 | .590 | .326 | .697 | CSR-F2 |
| CSR-F5 | .671–.792 | .790 | .557 | .786 | CSR-F3 |
| CF-Alternatives | .433–.673 | .863 | .330 | .706 | CSR-F2 |
| CF-Control | .424–.690 | .821 | .400 | .545 | CSE |
| CSE | .703–.791 | .886 | .564 | .626 | Creativity-Interpersonal |
| Creativity-Interpersonal | .499–.722 | .720 | .397 | .735 | Creativity-Academic |
| Creativity-Academic | .582–.674 | .726 | .399 | .741 | Creativity-Performance |
| Creativity-Performance | .721–.802 | .860 | .607 | .741 | Creativity-Academic |
| Creativity-Scientific | .700–.882 | .885 | .660 | .721 | Creativity-Academic |
| Creativity-Visual Arts | .687–.728 | .797 | .495 | .606 | Creativity-Academic |

**Note.** CSR = creative self-regulation; CF = cognitive flexibility; CSE = creative self-efficacy; CR = composite reliability; AVE = average variance extracted; HTMT = heterotrait–monotrait ratio of correlations. CSR-F1 = Expectation of Uncertainty; CSR-F2 = Strategy Adjustment; CSR-F3 = Psychological Self-Adjustment; CSR-F4 = Willingness to Share and Present Creative Outcomes; CSR-F5 = Optimization and Refinement of Creative Solutions. Maximum HTMT refers to the largest HTMT coefficient between the focal factor and any other first-order factor in the hypothesized measurement model; the corresponding factor is reported in the final column. All maximum HTMT values were below the conservative threshold of .85, supporting discriminant validity among the first-order factors (Henseler et al., 2015).

Supplementary Table S2. Measurement Invariance of the 20-K-DOCS Across Junior and Senior High School Students

| Model | Free parameters | $\chi^2$ | df | CFI | TLI | RMSEA | 90% CI | SRMR |
|---|---|---|---|---|---|---|---|---|
| Configural | 140 | 816.185 | 320 | .937 | .926 | .051 | [.046, .055] | .048 |
| Metric | 125 | 858.568 | 335 | .934 | .925 | .051 | [.047, .055] | .052 |
| Scalar | 110 | 902.332 | 350 | .930 | .924 | .051 | [.047, .055] | .055 |
